# Associations between cognitive function and long‐term cognitive enhancement therapy: Insights from a 5‐year follow‐up, case study

**DOI:** 10.1002/alz.090721

**Published:** 2025-01-09

**Authors:** Stalo Zarouna, Antonia Tziannarou, Savvina Chrysostomou, Sotiria Moza

**Affiliations:** ^1^ Materia Group, Nicosia, Nicosia Cyprus; ^2^ Materia Group, Nicosia Cyprus; ^3^ Noesis Cognitive Center & Tech Solutions Ltd, Nicosia Cyprus

## Abstract

**Background:**

A 69‐year‐old retired businessman, born in 1954, with 12 years of education, had been participating in cognitive enhancement sessions for the past 5 years. His medical history included two ischemic strokes, left hemiplegia, as well as disturbances in the left visual field. This study aimed to examine the individual’s cognitive performance over the course of these 5 years, including the COVID‐19 pandemic period.

**Methods:**

Bi‐weekly, 45‐minute cognitive remediation sessions were performed for 1.5 years, transitioning to once weekly later on. Exercises included orientation (e.g., use of calendar, news, articles), language (e.g., repetition, fluency, fill the gaps), visuospatial (e.g., pattern completion, object localisation, tracking of moving objects), memory (e.g., stories/words/images, learning, consolidation and recall of information, reminiscence and old songs), executive (e.g., dual tasks, categorisation) and motor tasks (e.g., ball games).

**Results:**

At baseline (July 2018), the person scored 11/30 in the Montreal Cognitive Assessment (MoCA) with significant visuospatial, memory recall, and orientation difficulties. In October 2019 (after 59 sessions), the MoCA score improved to 15/30, with better performance in visuospatial tasks, calculation, delayed recall, and orientation. However, a re‐evaluation in May 2020, following the first wave of COVID‐19, during which sessions were paused, resulted in a score of 11/30. The lowest points were noted in orientation and delayed recall. Sessions continued at a reduced frequency due to COVID‐19 restrictions and medical complications (flu, serious respiratory and urinary tract infections, and hospitalisation) which also affected the fine motor skills of the right hand. Last evaluation, in September 2023, yielded a score of 12/30 points, showing a regain of orientation and calculation scores, but losses in visuospatial skills, naming and delayed recall compared to the 2019 evaluation.

**Discussion:**

Our findings suggest a connection between cognitive functionality and cognitive enhancement therapy, particularly in orientation, memory recall, and calculation tasks. Achieving impact appears to require sustained and long‐term engagement in personalised enhancement sessions, which can be significantly hindered by external obstacles, such as COVID‐19 restrictions and other health issues.

